# Prescribing Druggists

**Published:** 1886-08

**Authors:** 


### *PRESCRIBING DRUGGISTS.*

All physicians practicing in cities are painfully aware of the great number of patients who come under their care after being treated by druggists. The great majority of patients with venereal diseases are treated, at least in the early stages of their trouble, by the drug clerk, and only call on the physician when obliged to take to their beds, or when their malady has reached a chronic stage, with various complications. The druggist prescribes for many other acute diseases in their early stages. We have been called to write death certificates for infants with diarrhœal disease, who have been dosed by the druggist for a week. Many druggists prepare and advertise a number of remedies (?) for numerous ills of suffering humanity. There are Blank's mixtures, Nos. 1, 2 and 3, for gonorrhœa; Blank's cough cure, for all respiratory affections; Blank's rheumatic remedy, for all aches and pains of muscle and joint; Blank's cholera cure, for all intestinal diseases, etc. These are openly advertised and prescribed, in addition to an occasional prescription stolen from some of their medical patrons. Many druggists, moreover, sell morphine and other poisons with the greatest impunity—although they utterly disregard the law regulating the sale of poisons. Within the past month, we have stood by the death-bed of a patient to whom a druggist had sold a dozen half grain morphine pills. They looked small. Patient swallowed six at one dose. He now rests with his fathers, and his widow takes in plain sewing. We have given a few doses of morphine hypodermically to a man affected with neuralgia. A druggist tells him to buy a hypodermic syringe, and makes him up a solution of morphine, and now he is about ready for the insane asylum.

These are a few illustrations of the harm done to patients by the prescribing druggists. Physicians can easily give examples ad infinitum. The physician is often injured by receiving those cases which have been treated by the druggist. Second-hand patients are seldom desirable, especially when, as is often the case, they have been ruined by the drug-store treatment. Moreover, the druggist himself is eventually harmed by such a course. Physicians are forced to dispense their own medicines, or, at least, to warn them against patronizing the prescribing druggist.

Various causes have led to this evil; as the *Southern California Practitioner* puts it, "the fact that the physician does not dispense his own medicines, and the excessive cost of the drugs as furnished by the druggist to fill the prescription which the physician has written. Many a person, in limited circumstances, says, I cannot afford to pay the physician for his advice and then pay the druggist for his medicines. Which shall I choose? The advice without the medicine will be of no avail, while some medicine without the advice might help me, and, as the druggist must know what physicians are in the habit of prescribing for similar complaints, I will try him first, and if I receive no benefit, will then seek medical advice. And so the sick man seeks the nearest drug store, and the druggist *prescribes over the counter.* But an important fact is overlooked by the sick man, and ignored by the druggist, viz.: that to treat disease, something more is needed than a knowledge of drugs and a haphazard guess at the name of the disease. Back of it all lie the knowledge of anatomy, of physiology, of the pathology of diseased action, the clinical training, the sharpened powers of observation; in other words, the special training of the physician."

To correct this practice, we would recommend, first, the enforcing of the law relative to the sale of poisons, and this should be attended to by the sanitary police, the board of health. Secondly, the censors of the medical societies should enforce the laws regulating the practice of medicine, thus preventing "*counter prescribing*," as it has been termed; for the prescribing druggist is nothing more nor less than an illegal practitioner, a quack. Lastly, the medical profession itself can do much to counteract this growing evil. The physician should give his patronage to those apothecaries who do a strictly legitimate business, and if such cannot be found, dispense his own medicines, thus severing all connection with the quack druggists. According to an Italian proverb, "Those who sleep with dogs, will catch fleas," so the physician who patronizes a quack druggist will come to harm, and the honor and dignity of his calling will suffer.

